# Estimated plasma volume status as a simple and accessible predictor of 28-day mortality in septic shock: insights from a retrospective study of the MIMIC-IV database

**DOI:** 10.3389/fmed.2024.1416396

**Published:** 2024-06-06

**Authors:** Beijun Gao, Rongping Chen, Hua Zhao, Hongmin Zhang, Xiaoting Wang, Dawei Liu

**Affiliations:** ^1^Department of Critical Care Medicine, Peking Union Medical College, Chinese Academy of Medical Sciences, Peking Union Medical College Hospital, Beijing, China; ^2^Department of Health Care, Peking Union Medical College, Chinese Academy of Medical Sciences, Peking Union Medical College Hospital, Beijing, China

**Keywords:** estimated plasma volume status, septic shock, fluid therapy, 28-day mortality, MIMIC-IV database

## Abstract

**Background:**

Assessing volume status in septic shock patients is crucial for tailored fluid resuscitation. Estimated plasma volume status (ePVS) has emerged as a simple and effective tool for evaluating patient volume status. However, the prognostic value of ePVS in septic shock patients remains underexplored.

**Methods:**

The study cohort consisted of septic shock patients admitted to the ICU, sourced from the MIMIC-IV database. Patients were categorized into two groups based on 28-day survival outcomes, and their baseline characteristics were compared. According to the ePVS (6.52 dL/g) with a hazard ratio of 1 in the restricted cubic spline (RCS) analysis, patients were further divided into high and low ePVS groups. A multivariable Cox regression model was utilized to evaluate the association between ePVS and 28-day mortality rate. The Kaplan–Meier survival curve was plotted, and all-cause mortality was compared between the high and low groups using the log-rank test.

**Results:**

A total of 7,607 septic shock patients were included in the study, among whom 2,144 (28.2%) died within 28 days. A J-shaped relationship was observed between ePVS at ICU admission and 28-day mortality, with an increase in mortality risk noted when ePVS exceeded 6.52 dL/g. The high ePVS group exhibited notably higher mortality rates compared to the low ePVS group (28-day mortality: 26.2% vs. 30.2%; 90-day mortality: 35% vs. 42.3%). After adjustment for confounding factors, ePVS greater than 6.52 dL/g independently correlated with an increased risk of 28-day mortality (HR: 1.20, 95% CI: 1.10–1.31, *p* < 0.001) and 90-day mortality (HR: 1.25, 95% CI: 1.15–1.35, *p* < 0.001). Kaplan–Meier curves demonstrated a heightened risk of mortality associated with ePVS values exceeding 6.52 dL/g.

**Conclusion:**

A J-shaped association was observed between ePVS and 28-day mortality in septic shock patients, with higher ePVS levels associated with increased risk of mortality.

## 1 Introduction

Septic shock is a form of circulatory failure characterized by a combination of mechanisms including hypovolemia, vascular tone depression, cardiac dysfunction, and disturbances in microcirculation (1). Fluid infusion is the most used treatment method in clinical practice (2). The purpose of fluid infusion is to increase cardiac output and improve tissue perfusion. However, in practice, fluid infusion can yield different outcomes (3, 4). Sepsis reduces vascular tone through various mechanisms, resulting not only in arterial hypotension due to vasodilation but also in venous dilation, altered blood flow distribution, and microcirculatory dysfunction (5). However, reduced venous tone increases unstressed volume, leading to venous return impairment, causing an increase in total body volume but failing to enhance venous return (6). Therefore, the effect of intravenous fluid infusion is difficult to maintain and can cause subsequent damage, leading to poor prognosis (7). We hypothesize that the total vascular volume in septic patients can reflect the extent of venous dilation in sepsis.

The traditional Strauss et al. formula, developed in 1951, utilizes an equation based on hematocrit and hemoglobin to provide estimations of plasma volume status (ePVS) (8). In 2015, Duarte et al. introduced a single time-point ‘instantaneous’-derived measurement of plasma volume for estimating PV derived from the Strauss formula (9). They found that, in cases of myocardial infarction complicated by heart failure (HF), ePVS, as an indicator of total vascular volume, holds significant prognostic value for early cardiovascular events beyond routine clinical evaluations.

ePVS offers a straightforward method to estimate plasma volume. As a surrogate marker for total vascular volume, it has been validated for its reliability, with multiple studies demonstrating its independent association with outcomes in various heart failure phenotypes (10–12). Moreover, ePVS has shown prognostic relevance in patients with acute respiratory distress syndrome and fever (13, 14). We hypothesize that in patients with septic shock, the total vascular volume will increase following fluid resuscitation due to the systemic vasodilation caused by the inflammatory response, and this increase in total vascular volume is related to the prognosis.

Despite the clinical utility and simplicity of ePVS, alongside its cost-effectiveness and efficiency, its adoption in clinical practice remains limited. This study aims to explore the impact of ePVS on the mortality of patients with septic shock, thereby contributing to the optimization of septic shock management.

## 2 Methods

### 2.1 The database

The Medical Information Mart for Intensive Care IV (MIMIC-IV version 2.2) database was utilized to gather the data for this investigation (15). The MIMIC-IV database collected clinical data on patients who visited Beth Israel Deaconess Medical Center (BIDMC) between 2008 and 2019. Access to the database is available for download upon completion of an authorized course on their official website. The author, Beijun Gao, has completed the accredited course, had database access, and oversaw data extraction (Record ID: 12338471). All patient information is hidden to protect their privacy.

### 2.2 Cohort information

#### 2.2.1 Selection of participants

Selection of patients diagnosed with septic shock in version 2.2 of the (MIMIC)-IV database. Septic shock is defined as patients who received appropriate fluid resuscitation but still require vasopressors to maintain mean arterial pressure (MAP) >65 mmHg, and serum lactate levels above 2.0 mmol/L (16), ICD9 and ICD10 codes are used to identify patients with septic shock. Inclusion criteria are as follows: first admission to the intensive care unit (ICU), age over 18 years, and ICU stay of at least 1 day. Exclusion criteria are: (i) multiple admissions; (ii) age < 18 years; (iii) during pregnancy and postpartum period; (iv) hospital stay <24 h; (v) Lack of data on hemoglobin levels and hematocrit values, or substantial baseline data absence.

#### 2.2.2 Variable extraction

We selected the first data point upon the target patient’s admission to the ICU. Baseline characteristics of patients include age, gender, weight, history of diabetes, history of hypertension, and history of malignant tumors. Vital signs data extracted from ICU patients include heart rate (HR), systolic blood pressure (SBP), diastolic blood pressure (DBP), mean arterial pressure (MAP), and respiratory rate (RR). Blood gas analysis indices include potassium (K^+^), sodium (Na^+^), anion gap, and lactate. Laboratory parameters include white blood cell count (WBC), platelet count (PLT), hematocrit (HCT), hemoglobin (HB), potassium (K^+^), sodium (Na^+^), anion gap, lactate, alanine aminotransferase (ALT), aspartate aminotransferase (AST), total bilirubin, prothrombin time (PT), international normalized ratio (INR), blood urea nitrogen (BUN) and creatinine (Cr). Intervention measures include ventilation, continuous renal replacement therapy (CRRT), and vasopressors. Vasopressor use is defined as the administration of norepinephrine, epinephrine, dopamine, dobutamine, or vasopressin during the first day of ICU hospitalization. Additionally, Charlson comorbidity index, Sequential Organ Failure Assessment (SOFA) score, Acute Physiology And Chronic Health Evaluation (APACHE) score, and worst renal function stage during hospitalization based on the Kidney Disease: Improving Global Outcomes (KDIGO) AKI Guideline Work Group criteria were calculated for each patient (17).

### 2.3 Evaluation of ePVS

The Duarte formula incorporating hematocrit and hemoglobin was utilized as follows (7):

$$\mathrm{ePVS}\left(\mathrm{dL}/g\right)=\left(100-\mathrm{hematocrit}\;\left(\%\right)\right)/\mathrm{hemoglobin}\left(g/\mathrm{dL}\right)$$

### 2.4 Grouping and study endpoints

Based on the 28-day follow-up outcomes, enrolled patients were categorized into the survival group (*n* = 5,463) and the death group (*n* = 2,144). Additionally, patients were further stratified into a high ePVS group (*n* = 3,789) and a low ePVS group (*n* = 3,818) based on the ePVS value (6.52 dL/g), which was determined through restricted cubic spline (RCS) analysis, as described later in this study. The primary outcome assessed in this study was 28-day mortality. The secondary outcomes included 90-day mortality, duration of ICU stay, and occurrence of acute kidney injury.

### 2.5 Statistical analysis

Data management procedures were implemented to address missing data issues. Cases with severely missing data, exceeding 20% of the dataset, were excluded from the analysis (Supplementary Table S1 for details on missing data). Acceptable missing data were imputed using the multiple imputation method with random forests, implemented in the R software package (18). Continuous variables underwent an initial assessment for normal distribution. Those adhering to a normal distribution were summarized as mean (standard deviation) and analyzed using the *t*-test method. Alternatively, continuous variables not conforming to a normal distribution were presented as median (interquartile range) and analyzed using nonparametric methods (Mann–Whitney *U* test). Categorical data were summarized as frequencies and percentages and analyzed using the chi-square method.

To investigate the relationship between ePVS and 28-day all-cause mortality risk in patients with septic shock, RCS analysis was performed. Subsequently, a cut-off value of 6.52 dL/g for ePVS was determined based on the RCS analysis, stratifying patients into high and low ePVS groups. Univariate and multivariate Cox regression analyses were conducted to assess the independent association between increased ePVS and higher 28-day and 90-day all-cause mortality in patients with septic shock. Results were expressed as hazard ratios (HR) with 95% confidence intervals (CI). Model I analysis involved no adjustments for covariates. In Model II, adjustments were made for Age, Gender, and Weight. Model III further adjusted for SOFA score, Charlson Comorbidity Index, APACHE II score, SAPS II score, HR, SBP, DBP, MAP, RR, lactate, K^+^, Na^+^, anion gap, WBC, PLT, ALT, AST, total bilirubin, PT, INR, BUN, Cr, mechanical ventilation, CRRT, vasopressor use, metastatic cancer, DM, and HT. Kaplan–Meier curves were generated to visualize the survival probability between high and low ePVS groups, with comparison done using the log-rank test. Stratified analyses were conducted based on the variables Gender, Age (>65 vs. <=65), Ventilation, CRRT, and Vasopressor use. Calibration curve was also included to better substantiate the predictive value of ePVS. Data analysis was performed using R programming language version 4.2.0, with statistical significance set at a two-tailed *p*-value of <0.05.

## 3 Results

### 3.1 Demographics and baseline characteristics

**Figure 1:**
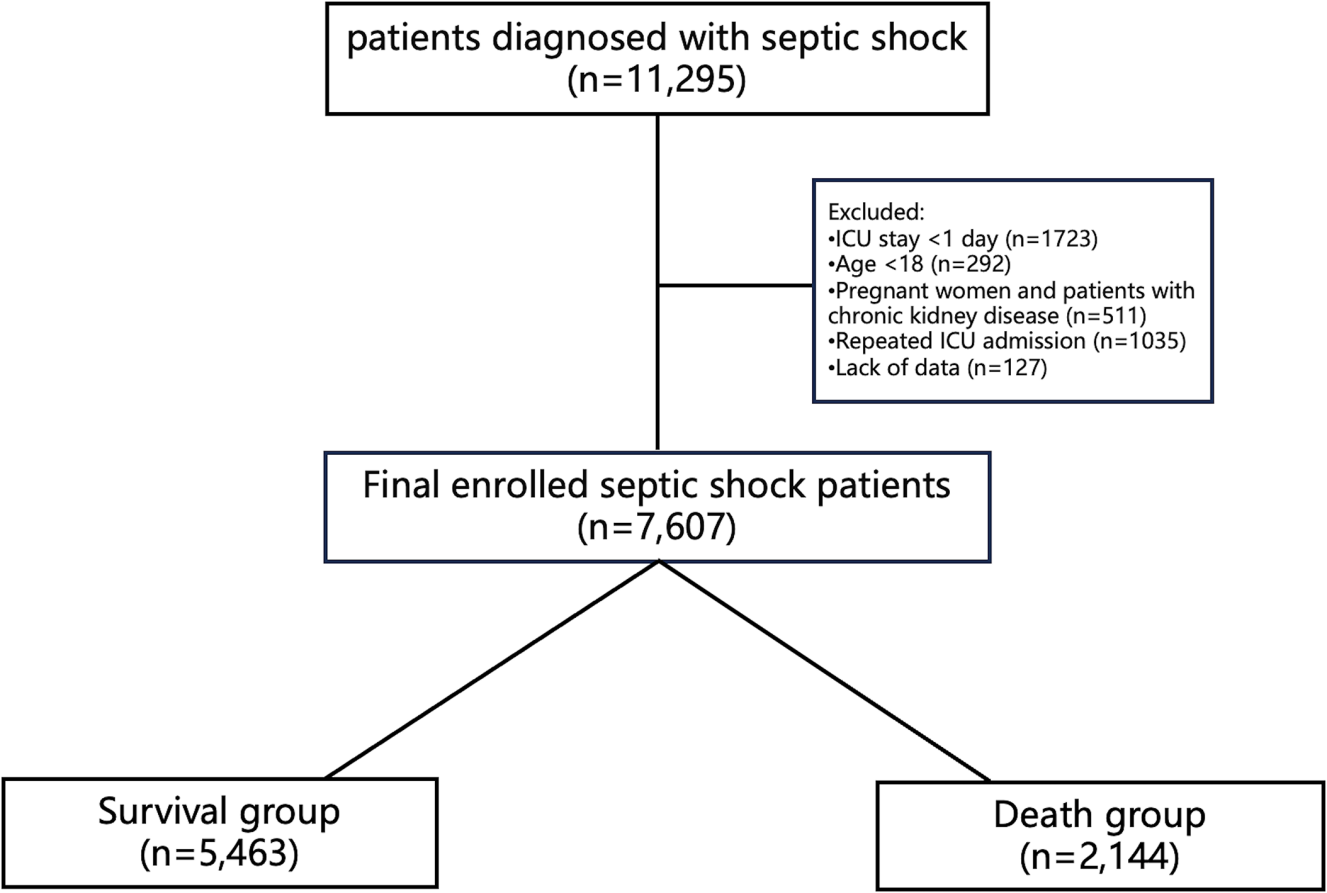
Flow chart of the study.

The study included 7,607 patients, with 5,463 (71.8%) surviving and 2,144 (28.2%) deceased (Figure 1). Deceased patients had a higher mean age (70.62 vs. 66.24 years) and lower body weight (79.60 vs. 82.63 kg) than survivors. Comorbidities like diabetes mellitus (DM: 3.7% vs. 2.8%) and hypertension (HT: 79.9% vs. 77.6%) were more prevalent in the deceased. Severity scores, including SOFA (9.60 vs. 6.99), Charlson (6.80 vs. 5.66), APACHE (73.59 vs. 55.57), and SAPS II (53.06 vs. 41.44), were higher in the deceased. Vital signs such as heart rate, systolic blood pressure, and respiratory rate were slightly elevated in the deceased. Laboratory findings showed deviations in lactate, potassium, WBC count, PLT count, liver function (ALT, AST, total bilirubin), and renal function (BUN, Cr) in the deceased, with significant differences compared to survivors. Mechanical ventilation, CRRT, and vasopressor usage were more frequent in the deceased cohort (Table 1).

**Table 1:** The characteristic of included subjects between different groups.

Variable	Level	Overall *n* = 7,607	Survival *n* = 5,463	Death *n* = 2,144	*p*-value
General information					
Age [mean (SD)]		67.47 (15.08)	66.24 (15.11)	70.62 (14.55)	<0.001
Gender (%)	Female	3,329 (43.8)	2,376 (43.5)	953 (44.4)	0.465
	Male	4,278 (56.2)	3,087 (56.5)	1,191 (55.6)	
Weight [mean (SD)]		81.78 (26.11)	82.63 (26.91)	79.60 (23.84)	<0.001
Comorbidities					
Metastatic cancer (%)	No	7,600 (99.9)	5,459 (99.9)	2,141 (99.9)	0.658
	Yes	7 (0.1)	4 (0.1)	3 (0.1)	
DM (%)	No	7,345 (96.6)	5,260 (96.3)	2,085 (97.2)	0.045
	Yes	262 (3.4)	203 (3.7)	59 (2.8)	
HT (%)	No	5,956 (78.3)	4,242 (77.6)	1,714 (79.9)	0.031
Score system					
SOFA [mean (SD)]		7.73 (3.79)	6.99 (3.41)	9.60 (4.04)	<0.001
Charlson [mean (SD)]		5.98 (2.89)	5.66 (2.83)	6.80 (2.87)	<0.001
APACHE II [mean (SD)]		60.65 (22.49)	55.57 (19.45)	73.59 (24.44)	<0.001
SAPSII [mean (SD)]		44.72 (14.73)	41.44 (13.20)	53.06 (15.11)	<0.001
Vital signs					
HR [mean (SD)]		111.40 (22.44)	110.14 (22.11)	114.61 (22.97)	<0.001
SBP [mean (SD)]		141.26 (22.75)	141.93 (22.51)	139.55 (23.28)	<0.001
DBP [mean (SD)]		86.92 (21.07)	87.14 (20.67)	86.38 (22.04)	0.159
MAP [mean (SD)]		103.52 (28.64)	103.40 (27.40)	103.83 (31.58)	0.557
RR [mean (SD)]		30.14 (6.96)	29.80 (6.93)	30.99 (6.98)	<0.001
Laboratory results					
ePVS [mean (SD)]		6.64 (1.80)	6.58 (1.77)	6.79 (1.85)	<0.001
Lac [mean (SD)]		3.21 (2.77)	2.78 (2.14)	4.31 (3.74)	<0.001
Potassium [mean (SD)]		4.73 (0.96)	4.68 (0.96)	4.83 (0.95)	<0.001
Sodium [mean (SD)]		139.71 (5.89)	139.68 (5.57)	139.81 (6.65)	0.392
Anion gap [mean (SD)]		18.41 (5.77)	17.77 (5.34)	20.06 (6.45)	<0.001
WBC [mean (SD)]		17.13 (12.56)	16.54 (10.68)	18.65 (16.30)	<0.001
PLT [mean (SD)]		230.71 (143.75)	236.69 (144.20)	215.46 (141.49)	<0.001
Hemoglobin [mean (SD)]		10.63 (2.07)	10.69 (2.06)	10.45 (2.07)	<0.001
ALT (median [IQR])		43.00 [20.00, 99.00]	43.00 [20.00, 93.97]	43.00 [20.00, 112.35]	0.042
AST (median [IQR])		71.00 [31.00, 152.00]	67.00 [29.00, 139.16]	82.00 [35.00, 194.25]	<0.001
Total bilirubin (median [IQR])		1.20 [0.50, 2.68]	1.20 [0.50, 2.50]	1.40 [0.60, 3.50]	<0.001
PT [mean (SD)]		21.51 (15.45)	20.26 (13.86)	24.72 (18.52)	<0.001
INR [mean (SD)]		1.99 (1.46)	1.87 (1.32)	2.29 (1.75)	<0.001
Bun [mean (SD)]		40.36 (28.36)	37.60 (26.84)	47.38 (30.83)	<0.001
Cr [mean (SD)]		2.21 (1.98)	2.13 (2.04)	2.41 (1.80)	<0.001
Treatment					
Ventilation (%)	No	4,472 (58.8)	3,403 (62.3)	1,069 (49.9)	<0.001
	Yes	3,135 (41.2)	2,060 (37.7)	1,075 (50.1)	
CRRT (%)	No	6,575 (86.4)	4,930 (90.2)	1,645 (76.7)	<0.001
	Yes	1,032 (13.6)	533 (9.8)	499 (23.3)	
Vasopressor (%)	No	5,668 (74.5)	4,532 (83.0)	1,136 (53.0)	<0.001
	Yes	1,939 (25.5)	931 (17.0)	1,008 (47.0)	
Outcomes					
AKI stage (%)	0	1,475 (19.4)	1,331 (24.4)	144 (6.7)	<0.001
	1	949 (12.5)	779 (14.3)	170 (7.9)	
	2	2,177 (28.6)	1,728 (31.6)	449 (20.9)	
	3	3,006 (39.5)	1,625 (29.7)	1,381 (64.4)	
Losicu [mean (SD)]		6.89 (8.08)	7.00 (8.89)	6.61 (5.47)	0.059

Results are expressed as mean (SD), median [IQR] or *n* (%). DM, Diabetes Mellitus; HT, Hypertension; SOFA, Sequential Organ Failure Assessment score; Charlson, comorbidity index; APACHE II, Acute Physiology And Chronic Health Evaluation II; SASPII, Severe Acute Pancreatitis Score II; HR, Heart Rate; SBP, Systolic Blood Pressure; DBP, Diastolic Blood Pressure; MAP, Mean Arterial Pressure; RR, Respiratory Rate; ePVS, Estimated plasma volume status; Lac, Lactate; WBC, White Blood Cell count; PLT, Platelet count; ALT, Alanine Aminotransferase; AST, Aspartate Aminotransferase; PT, Prothrombin Time; INR, International Normalized Ratio; BUN, Blood Urea Nitrogen; Cr, Creatinine; CRRT, Continuous Renal Replacement Therapy; AKI, Acute Kidney Injury; Losicu, Length of ICU Stay.

### 3.2 Restricted cubic spline (RCS) analysis between ePVS level and 28-day mortality

**Figure 2:**
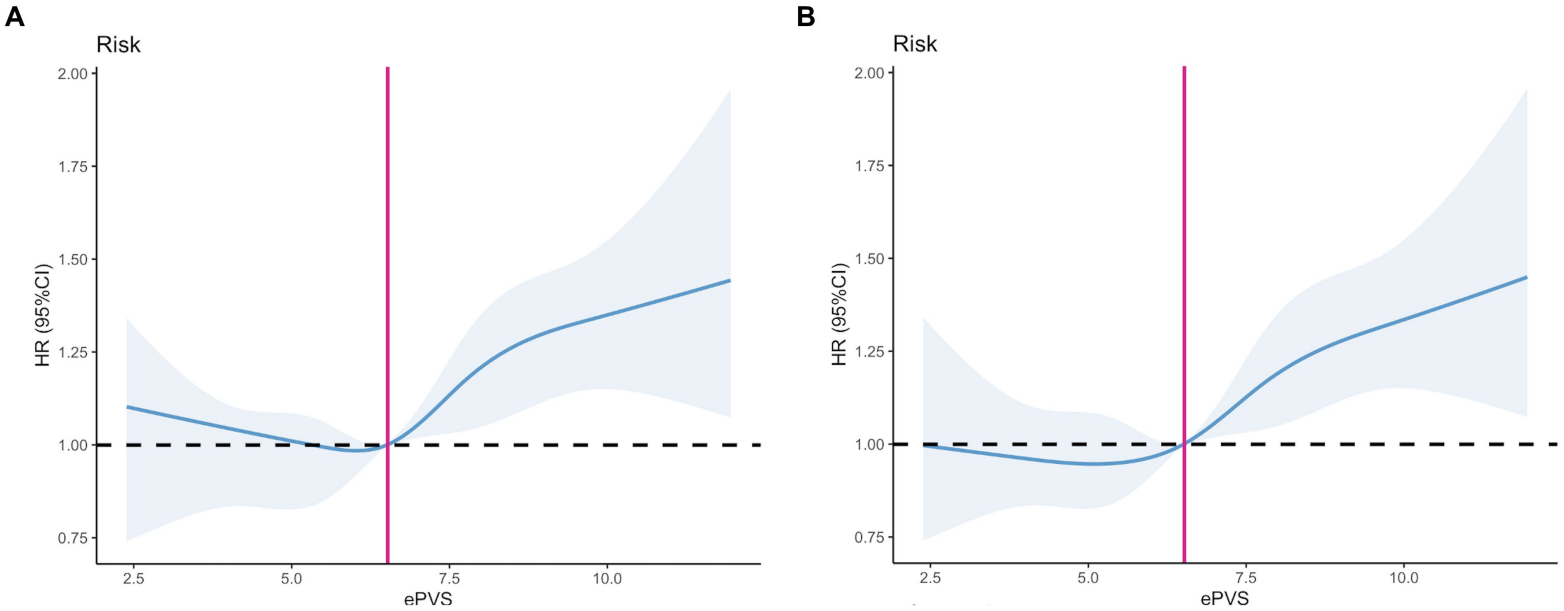
Restricted cubic spline (RCS). **(A)** Unadjusted model. **(B)** Adjusted model: Adjusted for Age, Gender, Weight, SOFA score, Charlson Comorbidity Index, APACHE II score, SAPS II score, Heart Rate (HR), Systolic Blood Pressure (SBP), Diastolic Blood Pressure (DBP), Mean Arterial Pressure (MAP), Respiratory Rate (RR), Lactate, Potassium, Sodium, Anion Gap, White Blood Cell (WBC) count, Platelet (PLT) count, Alanine Aminotransferase (ALT), Aspartate Aminotransferase (AST), Total Bilirubin, Prothrombin Time (PT), International Normalized Ratio (INR), Blood Urea Nitrogen (BUN), Creatinine (Cr), Mechanical Ventilation, Continuous Renal Replacement Therapy (CRRT), Vasopressor Use, Metastatic Cancer, Diabetes Mellitus (DM), and Hypertension (HT).

The relationship between admission ePVS level and 28-day mortality demonstrates a nonlinear pattern. Figure 2A presents the restricted cubic spline (RCS) curve illustrating this relationship in patients with septic shock, without adjusting for potential confounders. Upon adjustment for potential confounders, as shown in Figure 2B, both figures indicate that the cutoff value of ePVS was identified as 6.52 dL/g, corresponding to a hazard ratio of 1. Significant escalation in mortality risk was observed when ePVS exceeded 6.52 dL/g.

### 3.3 Outcomes by ePVS level in patients with septic shock

Table 2 presents outcomes based on ePVS levels in patients with septic shock. The 28-day and 90-day all-cause mortality rates were 28.2 and 38.6%, respectively. Notably, the 28-day mortality rate in the high ePVS group (30.2%) was significantly elevated compared to the low ePVS group (26.2%, *p* < 0.001). Similarly, the 90-day mortality rate in the high ePVS group (42.3%) was also higher than that in the low ePVS group (35%, *p* < 0.001). There was no significant difference in length of stay in the ICU between the two groups. Additionally, 39.5% of patients experienced stage 3 acute kidney injury (AKI), with a higher proportion observed in the high ePVS group (40.9%) compared to the low ePVS group (38.1%).

**Table 2:** The comparison of outcomes between the low ePVS group and high ePVS group.

Outcome	Level	Overall	Low ePVS ≤6.52 dL/g	High ePVS >6.52 dL/g	*p*-value
AKI stage (%)	0	1,475 (19.4)	731 (19.1)	744 (19.6)	0.014
	1	949 (12.5)	478 (12.5)	471 (12.4)	
	2	2,177 (28.6)	1,153 (30.2)	1,024 (27.0)	
	3	3,006 (39.5)	1,456 (38.1)	1,550 (40.9)	
Losicu [mean (SD)]		6.89 (8.08)	7.06 (8.53)	6.72 (7.59)	0.067
28-day mortality (%)		2,144 (28.2)	1,000 (26.2)	1,144 (30.2)	<0.001
90-day mortality (%)		2,940 (38.6)	1,336 (35.0)	1,604 (42.3)	<0.001

Results are expressed as mean (SD), *n* (%). ePVS, Estimated plasma volume status; AKI, Acute Kidney Injury; Losicu, Length of ICU Stay.

### 3.4 Survival analysis

**Figure 3:**
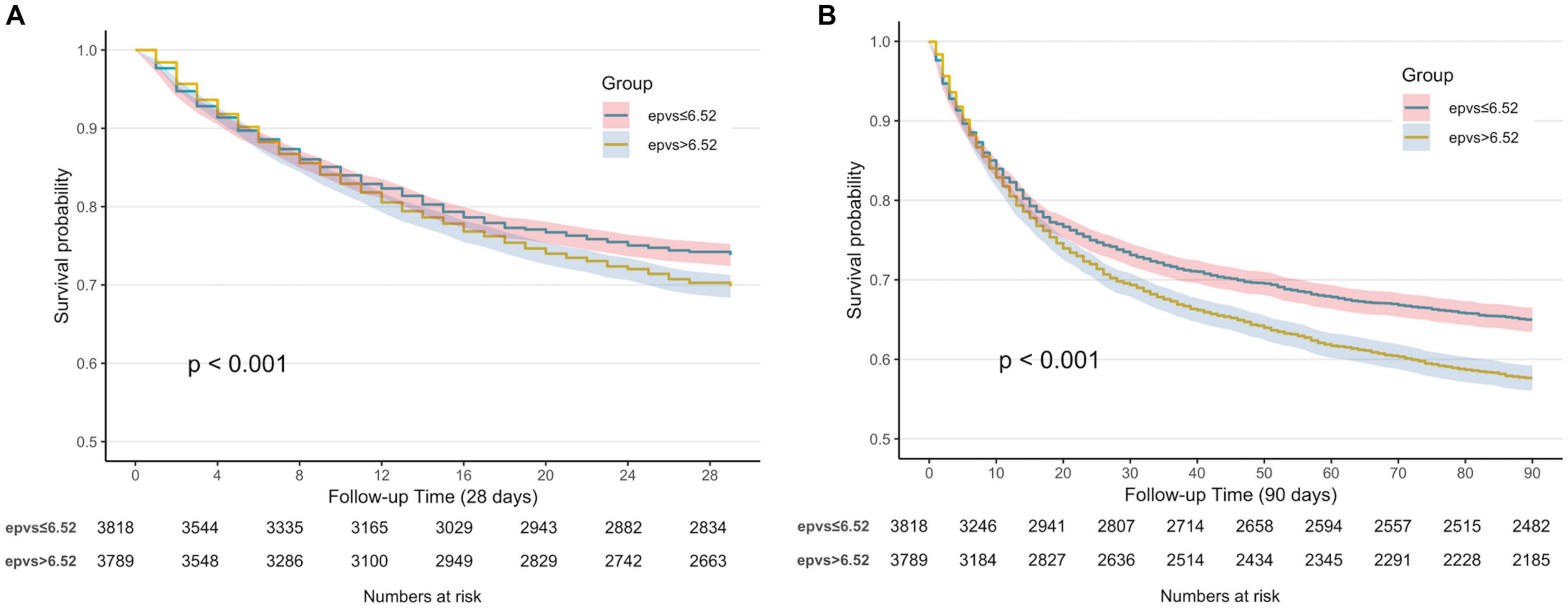
**(A)** Kaplan–Meier survival curve of 28-day cumulative survival rate for low and high ePVS groups. **(B)** Kaplan–Meier survival curve of 90-day cumulative survival rate for low and high ePVS groups.

The Kaplan–Meier curve showed that the 28-day and 90-day cumulative survival rates were lower in the high ePVS group than that in the low ePVS group (log-rank test, *χ*^2^ = 12.6, *p* < 0.001; *χ*^2^ = 35.9, *p* < 0.001) (Figures 3A,B).

### 3.5 Correlation between ePVS and all-cause mortality

**Figure 4:**
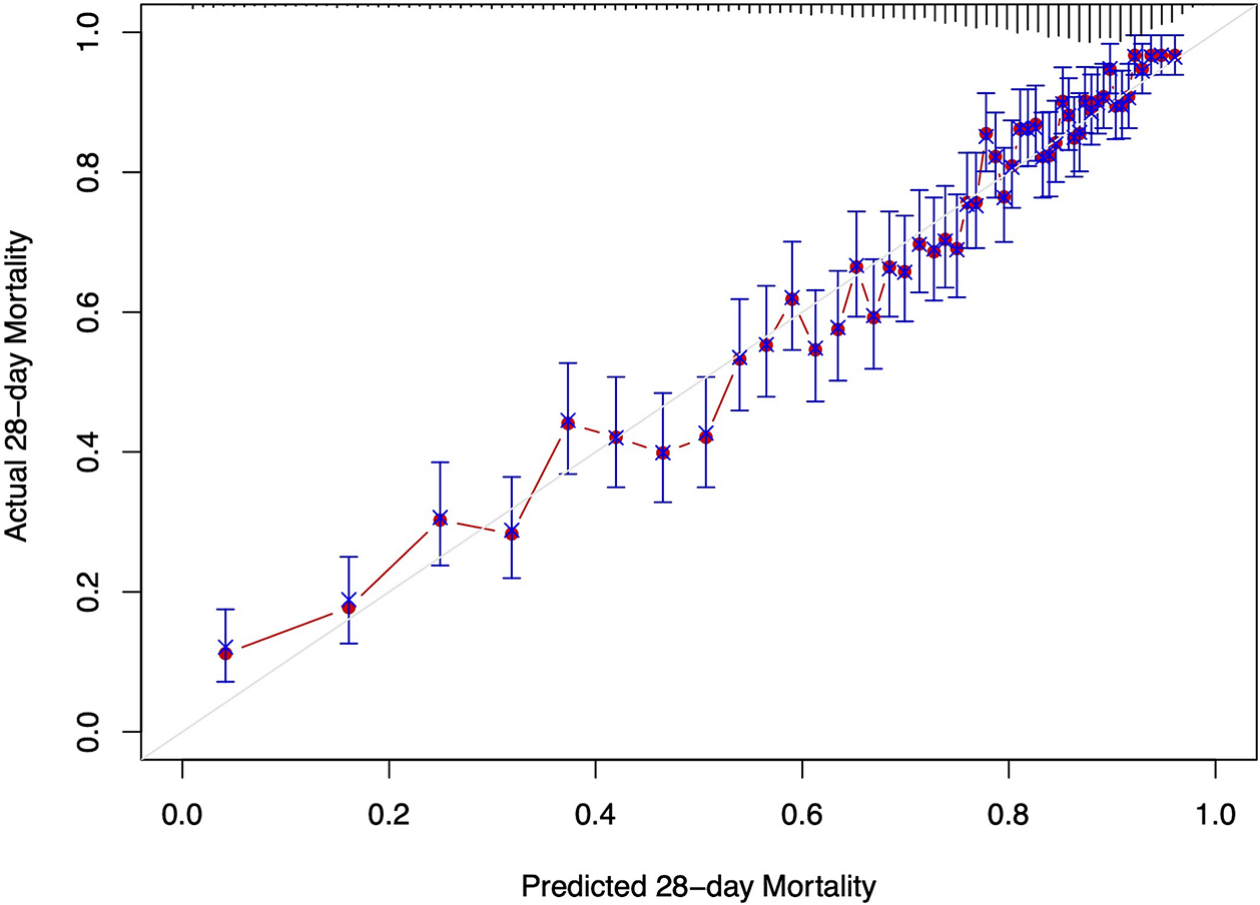
Calibration curve for EPVS in predicting 28-day mortality. The *x*-axis represents the predicted 28-day mortality based on ePVS measurements, while the *y*-axis shows the actual 28-day mortality. The blue crosses compare the predicted values with the actual outcomes, and the error bars indicate the confidence intervals of the predictions. The red line shows the trend between predicted and actual values.

The Cox regression models presented in Table 3 demonstrate the relationship between ePVS levels and the risk of 28-day and 90-day mortality. In the unadjusted model (Model I), ePVS as a continuous variable shows a significant positive correlation with 28-day and 90-day all-cause mortality (HR 1.05, 95% CI 1.03–1.08, *p* < 0.001 and HR 1.08, 95% CI 1.05–1.10, *p* < 0.001). When ePVS is categorized, higher levels (>6.52) are associated with increased 28-day and 90-day all-cause mortality (HR 1.17, 95% CI 1.07–1.27, *p* < 0.001 and HR 1.25, 95% CI 1.16–1.34, *p* < 0.001). In Model II, ePVS as a continuous variable remains positively correlated with 28-day and 90-day mortality. After categorization, high ePVS (>6.52) is still linked to increased 28-day mortality (HR 1.18, 95% CI 1.08–1.29, *p* < 0.001) and 90-day mortality (HR 1.26, 95% CI 1.17–1.36, *p* < 0.001). Model III results indicate that higher ePVS is an independent risk factor for adverse outcomes in patients with septic shock, with significantly higher 28-day (HR 1.20, 95% CI 1.10–1.31, *p* < 0.001) and 90-day (HR 1.25, 95% CI 1.15–1.35, *p* < 0.001) all-cause mortality rates in the high ePVS group. The calibration curve (Figure 4) demonstrates that the ePVS model reliably predicts 28-day mortality in septic shock patients, with predicted probabilities closely matching observed outcomes. This supports the potential utility of ePVS as a prognostic tool in clinical settings.

**Table 3:** ePVS levels and all-cause in-hospital mortality of septic shock.

	Model I, HR 95%CI, *p* value	Model II, HR, 95%CI, *p* value	Model III, HR, 95%CI, *p* value
28-day mortality			
ePVS (continuous variable)	1.05 (1.03–1.08, *p* < 0.001)	1.06 (1.03–1.08, *p* < 0.001)	1.07 (1.04–1.09, *p* < 0.001)
ePVS [Categorical variables (quartile)]			
≤6.52	Ref	Ref	Ref
>6.52	1.17 (1.07–1.27, *p* < 0.001)	1.18 (1.08–1.29, *p* < 0.001)	1.20 (1.10–1.31, *p* < 0.001)
P for trend	*p* < 0.001	*p* < 0.001	*p* < 0.001
90-day mortality			
ePVS (continuous variable)	1.08 (1.05–1.10, *p* < 0.001)	1.08 (1.06–1.10, *p* < 0.001)	1.08 (1.06–1.10, *p* < 0.001)
ePVS [Categorical variables (quartile)]			
≤6.52	Ref	Ref	Ref
>6.52	1.25 (1.16–1.34, *p* < 0.001)	1.26 (1.17–1.36, *p* < 0.001)	1.25 (1.15–1.35, *p* < 0.001)
P for trend	*p* < 0.001	*p* < 0.001	*p* < 0.001

HR, hazard ratio; CI, confidence interval; Ref, reference.

Model I: No adjustments.

Model II: Adjusted for Age, Gender, and Weight.

Model III: In addition to Model II adjustments, further adjustments were made for SOFA score, Charlson Comorbidity Index, APACHE II score, SAPS II score, Heart Rate (HR), Systolic Blood Pressure (SBP), Diastolic Blood Pressure (DBP), Mean Arterial Pressure (MAP), Respiratory Rate (RR), Lactate, Potassium, Sodium, Anion Gap, White Blood Cell (WBC) count, Platelet (PLT) count, Alanine Aminotransferase (ALT), Aspartate Aminotransferase (AST), Total Bilirubin, Prothrombin Time (PT), International Normalized Ratio (INR), Blood Urea Nitrogen (BUN), Creatinine (Cr), Mechanical Ventilation, Continuous Renal Replacement Therapy (CRRT), Vasopressor Use, Metastatic Cancer, Diabetes Mellitus (DM), and Hypertension (HT).

### 3.6 Subgroup analyses

**Figure 5:**
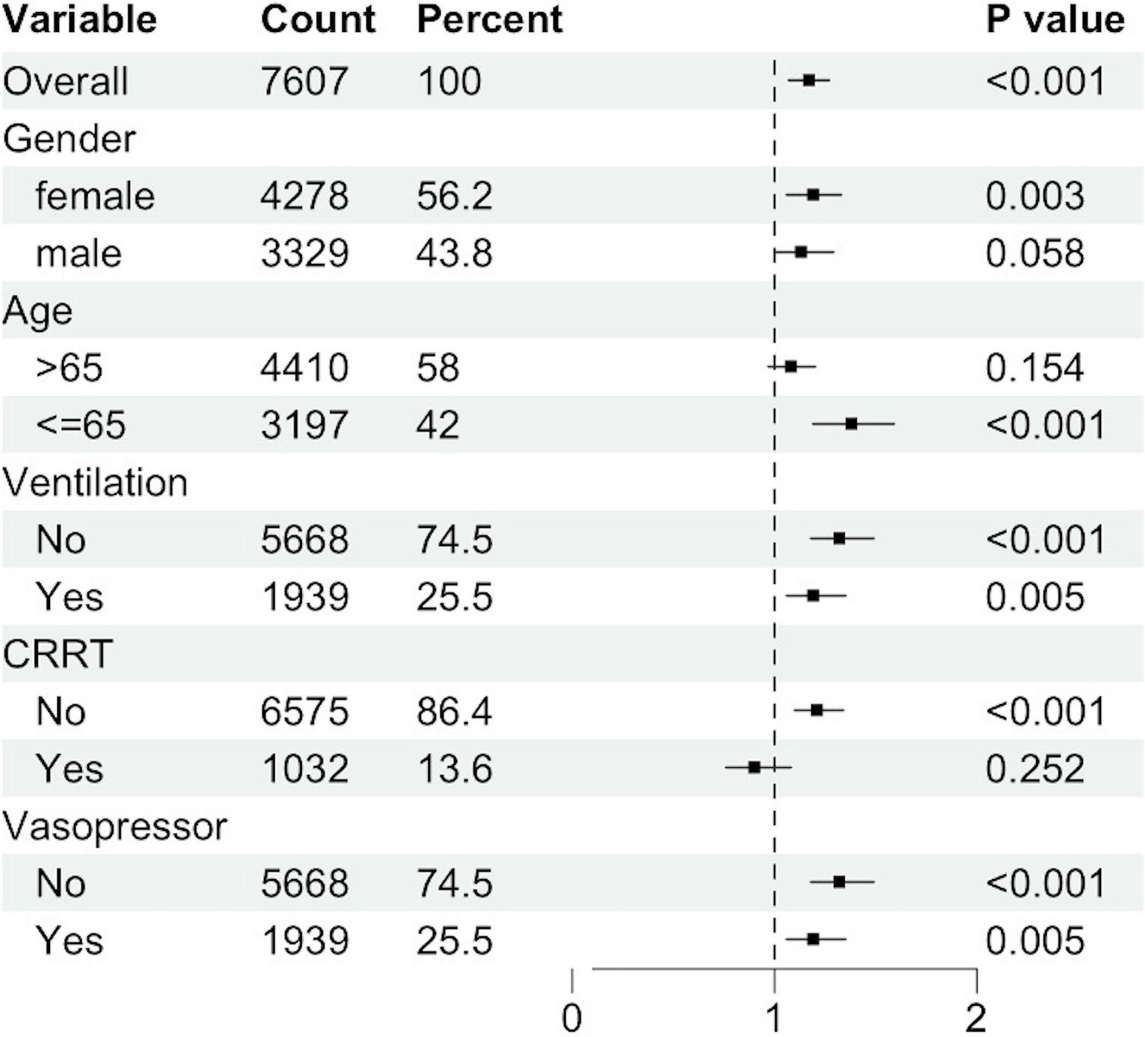
Subgroup analysis of the association between ePVS and 28-day mortality.

Subgroup analyses revealed significant associations within specific strata (Figure 5). Female gender exhibited a heightened risk for the primary outcome, with a hazard ratio (HR) of 1.19 (95% CI: 1.06–1.33), compared to male patients (HR: 1.13, 95% CI: 1.00–1.29). Patients aged 65 years or younger demonstrated an HR of 1.38 (95% CI: 1.19–1.59). Non-ventilated patients were at a significantly increased risk for the primary outcome (HR: 1.32, 95% CI: 1.18–1.49) compared to ventilated patients (HR: 1.19, 95% CI: 1.06–1.35). The HR for patients not receiving Continuous Renal Replacement Therapy (CRRT) was 1.21 (95% CI: 1.10–1.34), whereas for those receiving CRRT, the HR was 0.90 (95% CI, 0.76 to 1.08). Non-vasopressor use was associated with an elevated risk of the primary outcome (HR: 1.32, 95% CI: 1.18–1.49) compared to vasopressor use (HR: 1.19, 95% CI: 1.06–1.35).

## 4 Discussion

In this retrospective cohort study, we analyzed 7,607 ICU patients with septic shock, finding that 2,144 (28.2%) succumbed within 28 days. We identified a J-shaped relationship between estimated plasma volume status (ePVS) at ICU admission and 28-day mortality, with a significant increase in mortality risk when ePVS exceeded 6.52 dL/g. Multivariable Cox regression analysis showed a positive correlation between baseline ePVS above 6.52 dL/g and the risk of death at both 28 and 90 days. Kaplan–Meier curves demonstrated an increased risk of in-hospital mortality for ePVS values over 6.52 dL/g. Calibration curves further confirmed the predictive value of ePVS. Our findings suggest that ePVS, being readily accessible, holds promise as a prognostic tool for patients with septic shock.

ePVS was initially utilized in heart failure patients, with Duarte et al. leading its application by demonstrating its predictive value for early cardiovascular events in heart failure complicating myocardial infarction (9). Subsequent studies have consistently revealed its association with early clinical outcomes of decompensated heart failure and its potential to enhance risk stratification for heart failure (19). Another investigation conducted among US adults unveiled a robust correlation between increasing ePVS and elevated rates of all-cause mortality, cardiovascular mortality, and cancer-related mortality (4). In the emergency department, ePVS has been confirmed to be associated with the diagnosis and prognosis of dyspneic patients (20), as well as in ARDS (14), and also with febrile emergency department patients (21). Furthermore, ePVS has demonstrated a correlation with the severity of lower limb arterial disease and clinical outcomes (22).

In our study involving patients with septic shock, we found that ePVS levels were higher compared to those with cardiovascular diseases. Our cutoff value was set at 6.52 dL/g. This is an intriguing result, as in patients with acute myocardial infarction, an ePVS ≥5.28 mL/g emerged as a risk factor for in-hospital mortality and was associated with an elevated risk of 30-day mortality (23). However, in the report by Kim et al. (24), they investigated ICU patients with sepsis or septic shock. Their findings revealed an ePVS of 7.7 ± 2.1 dL/g, which stood out prominently, emphasizing a significant correlation between ePVS and the amount of intravenous fluid resuscitation in deceased patients. Additionally, they evaluated the utility of ePVS in predicting in-hospital mortality and identified a cutoff point of 7.09 dL/g. They observed a significant association between higher ePVS and increased in-hospital mortality (OR, 1.39; 95% CI, 1.04–1.85, *p* = 0.028). Gianni Turcato et al. conducted three studies on the application of ePVS in emergency department (ED) patients. Among 1,502 febrile patients in the emergency department, the median ePVS value in patients diagnosed with sepsis was 5.54 (4.43–6.51) dL/g, compared to a median ePVS value of 4.51 (3.89–5.24) dL/g in non-septic patients (*p* < 0.001). In multivariate analysis, an ePVS higher than 4.52 dL/g was associated with a odds ratio of 1.824 (95% CI 1.055–3.154, *p* = 0.030) for 30-day mortality (21). For emergency department patients diagnosed with sepsis, it was observed that the average ePVS among those surviving to 30 days was 5.19, while the average ePVS among those who died within 30 days was 5.74 (*p* = 0.004). ePVS emerged as an independent risk factor for 30-day mortality, with an adjusted odds ratio of 1.211 (95% CI 1.004–1.460, *p* = 0.045) (25). A recently published prospective study measured ePVS in 949 infected patients included in the study. The median ePVS value for patients who died within 30 days was higher than that of survivors (5.83 vs. 4.61, *p* < 0.001). Multivariate analysis demonstrated that ePVS, both in continuous and categorical forms around the median, was an independent risk factor for 30-day mortality even after adjusting for severity, comorbidity, and urgency (13).

We observed that ePVS levels were elevated in patients diagnosed with sepsis compared to those with fever, and notably higher in patients with septic shock. Our study revealed a significant increase in mortality risk when ePVS exceeded 6.52 dL/g. These differing cutoff values may be linked to the pathophysiology of septic shock. Primarily, a hallmark of sepsis is vascular paralysis, characterized by a decrease in arterial pressure and extensive venous dilation in both visceral and cutaneous vascular beds (26). Throughout the progression of sepsis, plasma volume does not decrease but rather increases the unstressed volume, thereby reducing venous return and cardiac output (1, 27). Secondly, the microcirculatory disturbances caused by systemic inflammatory response alter vascular permeability, irreversibly affecting the body’s volume regulation and the balance between interstitial and intravascular spaces (27). Additionally, early treatment for septic patients involves intravenous fluid resuscitation to restore tissue perfusion (28). Thus, as mentioned earlier, ePVS correlates with the volume of fluid administered before admission to the intensive care unit (24). The gradual accumulation of resuscitative fluids ultimately leads to intravascular congestion.

The association between ePVS and mortality in septic shock has been unequivocally established in our study. After multiple adjustments for variables, we consistently confirmed that an elevated ePVS serves as an independent risk factor for 28-day mortality in septic shock. One of the reasons for this correlation is the association between ePVS and venous congestion, which can serve as a marker of hemodynamic congestion. Research correlating ePVS with hemodynamic indices has shown a notable correlation between higher ePVS derived from the Duarte formula and higher E/e’ ratios. Interestingly, only in females, left ventricular end-diastolic pressure (LVEDP) is associated with ePVS (29). Moreover, in our subgroup analysis, we observed differences in ePVS between genders, indicating that the influence of gender on plasma volume regulation requires further investigation. While studies have suggested that ePVS is not correlated with pulmonary artery wedge pressure (PAWP) and intracardiac filling pressures (30), this may indirectly suggest that ePVS is more closely associated with the regulation of systemic venous beds and venous return rather than with cardiac function. It is essential to prioritize venous return function during fluid therapy. In summary, our study identified ePVS as an independent predictor of 28-day mortality in septic shock. Elevated ePVS levels may indicate the need for clinicians to prioritize venous return function during fluid therapy and to be vigilant about fluid redistribution in patients with septic shock, thereby assessing their volume status accordingly.

Our study has several limitations. Firstly, retrospective cohort studies inevitably entail biases. However, we attempted to adjust for potential confounders in our data analysis to minimize bias. Secondly, in our inclusion of patients with septic shock in the ICU, we did not extract information on fluid resuscitation in the emergency department, making it difficult to explore the relationship between fluid infusion and ePVS. Thirdly, we only selected ePVS at ICU admission. While we believe ePVS can serve as a continuous target variable to assess its impact on the prognosis of patients with septic shock, we plan to conduct further prospective studies to explore the continuous changes in ePVS and the prognosis of sepsis. Fourthly, although there is a correlation between the ePVS formula and actual plasma volume, this relationship needs to be validated against a gold standard (31). Fifthly, patients known to have chronic anemia were not excluded. Certain ePVS values may be altered due to these conditions. Lastly, due to limitations in the database, there were no echocardiographic data or hemodynamic data related to patient blood volume status and ePVS values in this dataset.

## 5 Conclusion

In patients with septic shock admitted to the ICU, there exists a J-shaped relationship between the first obtained ePVS values during routine blood tests and the 28-day mortality rate. When blood ePVS exceeds 6.52 dL/g, it is associated with an increased risk of mortality at both 28 and 90 days. However, prospective evidence is needed to confirm these clinical observations and to study the pathophysiological reasons behind elevated ePVS values. Nonetheless, high ePVS levels can serve as important indicators of the severity of illness in patients with septic shock.

## Data availability statement

The raw data supporting the conclusions of this article will be made available by the authors, without undue reservation.

## Ethics statement

The establishment of this database received approval from both the Massachusetts Institute of Technology (Cambridge, MA) and Beth Israel Deaconess Medical Center (Boston, MA), with consent obtained for the original data collection. The studies were conducted in accordance with the local legislation and institutional requirements. Written informed consent for participation was not required from the participants or the participants’ legal guardians/next of kin in accordance with the national legislation and institutional requirements.

## Author contributions

BG: Writing – original draft, Writing – review & editing. RC: Data curation, Writing – review & editing. HuZ: Data curation, Methodology, Writing – review & editing. HoZ: Conceptualization, Supervision, Writing – review & editing. XW: Supervision, Writing – review & editing. DL: Funding acquisition, Supervision, Writing – review & editing.
