# Supplementary material for: Estimated plasma volume status as a simple and accessible predictor of 28-day mortality in septic shock: insights from a retrospective study of the MIMIC-IV database
Source: Front Med (Lausanne). 2024 Jun 6;11:1416396. doi: 10.3389/fmed.2024.1416396 (PMC11187084; doi:10.3389/fmed.2024.1416396)
Supplement: Supplementary file 1 [file Table_1.docx]

Table S1 Missing number (%) for variables

| variable | Missing number | Percent% |
| --- | --- | --- |
| Age | 0 | 0.00% |
| Gender | 0 | 0.00% |
| Height | 3458 | 45.48% |
| Weight | 611 | 8.04% |
| Metastatic cancer | 0 | 0.00% |
| DM | 0 | 0.00% |
| HT | 0 | 0.00% |
| SOFA | 0 | 0.00% |
| Charlson | 0 | 0.00% |
| APACHE | 0 | 0.00% |
| SAPSII | 0 | 0.00% |
| HR | 5 | 0.07% |
| SBP | 30 | 0.39% |
| DBP | 30 | 0.39% |
| MAP | 6 | 0.08% |
| RR | 5 | 0.07% |
| P02 | 3068 | 40.35% |
| PC02 | 3068 | 40.35% |
| PH | 3069 | 40.36% |
| Lac | 1040 | 13.68% |
| Potassium | 11 | 0.14% |
| Sodium | 11 | 0.14% |
| Anion gap | 13 | 0.17% |
| WBC | 16 | 0.21% |
| PLT | 17 | 0.22% |
| Hematocrit | 0 | 0% |
| Hemoglobin | 0 | 0% |
| ALT | 1494 | 19.65% |
| AST | 1451 | 19.08% |
| Total bilirubin | 1491 | 19.61% |
| Albumin | 3008 | 39.56% |
| PT | 449 | 5.90% |
| INR | 449 | 5.90% |
| Bun | 7 | 0.09% |
| Cr | 7 | 0.09% |
| NTproBNP | 6865 | 90.28% |
| CVP | 5824 | 76.59% |
| Ventilation | 0 | 0.00% |
| CRRT | 0 | 0.00% |
| Vasopressor | 0 | 0.00% |
| AKI stage | 0 | 0.00% |
| Losicu | 0 | 0.00% |
